# Local and Regional Diversity Reveals Dispersal Limitation and Drift as Drivers for Groundwater Bacterial Communities from a Fractured Granite Formation

**DOI:** 10.3389/fmicb.2016.01933

**Published:** 2016-12-06

**Authors:** E. D. Beaton, Bradley S. Stevenson, Karen J. King-Sharp, Blake W. Stamps, Heather S. Nunn, Marilyne Stuart

**Affiliations:** ^1^Chalk River Laboratories, Canadian Nuclear Laboratories, Chalk RiverON, Canada; ^2^Department of Microbiology and Plant Biology, University of Oklahoma, NormanOK, USA

**Keywords:** fractured granite, subsurface, dispersal, selection, meta-community

## Abstract

Microorganisms found in terrestrial subsurface environments make up a large proportion of the Earth’s biomass. Biogeochemical cycles catalyzed by subsurface microbes have the potential to influence the speciation and transport of radionuclides managed in geological repositories. To gain insight on factors that constrain microbial processes within a formation with restricted groundwater flow we performed a meta-community analysis on groundwater collected from multiple discrete fractures underlying the Chalk River Laboratories site (located in Ontario, Canada). Bacterial taxa were numerically dominant in the groundwater. Although these were mainly uncultured, the closest cultivated representatives were from the phenotypically diverse Betaproteobacteria, Deltaproteobacteria, Bacteroidetes, Actinobacteria, Nitrospirae, and Firmicutes. Hundreds of taxa were identified but only a few were found in abundance (>1%) across all assemblages. The remainder of the taxa were low abundance. Within an ecological framework of selection, dispersal and drift, the local and regional diversity revealed fewer taxa within each assemblage relative to the meta-community, but the taxa that were present were more related than predicted by chance. The combination of dispersion at one phylogenetic depth and clustering at another phylogenetic depth suggest both niche (dispersion) and filtering (clustering) as drivers of local assembly. Distance decay of similarity reveals apparent biogeography of 1.5 km. Beta diversity revealed greater influence of selection at shallow sampling locations while the influences of dispersal limitation and randomness were greater at deeper sampling locations. Although selection has shaped each assemblage, the spatial scale of groundwater sampling favored detection of neutral processes over selective processes. Dispersal limitation between assemblages combined with local selection means the meta-community is subject to drift, and therefore, likely reflects the differential historical events that have influenced the current bacterial composition. Categorizing the study site into smaller regions of interest of more closely spaced fractures, or of potentially hydraulically connected fractures, might improve the resolution of an analysis to reveal environmental influences that have shaped these bacterial communities.

## Introduction

Biogeochemical cycles of the subsurface have the potential to influence the speciation and transport of radionuclides managed in geological repositories. There is limited knowledge on how microbial diversity relates to biogeochemical processes (Griebler and Lueders, 2009) such as the flow of energy through an ecosystem, and how elements like carbon are recycled. These processes involve metabolism and competition, where microorganisms act as catalysts and the available free energy supports a community sensing and responding to environmental changes (Lever, 2011; Jorgensen et al., 2012; Flynn et al., 2013; Algora et al., 2015). While these interactions can connect the supply of electron donor and acceptor compounds to abundant taxa, when viewed at multiple spatial scales, random factors like dispersal, speciation and extinction also influence diversity (Hubbell, 2001). Random processes, therefore, may also explain community dynamics irrespective of the available free energy. Taxa distributions between sampling locations, then, can stem from a combination of competition, environmental constraints, differences in dispersal among a regional pool of taxa and drift due to dispersal limitation.

In the study of subsurface environments, fractured granite formations represent an ecosystem with circuitous hydraulic flow paths that restrict both the flow direction and flow rate of the groundwater; these flow paths reflect complex local and regional recharge. Dispersal limitation, therefore, may obscure the detection of *in situ* biogeochemical processes except at sufficiently closely spaced sampling locations relative to recharge and discharge. Diverse microbial communities within these formations (Jain et al., 1997; Haveman et al., 1999; Sahl et al., 2008; Itävaara et al., 2011; Thompson et al., 2011; Hallbeck and Pedersen, 2012; Nyyssönen et al., 2012, 2014) display activities for nitrate, iron and sulfate reduction (Pedersen, 1996; Jain et al., 1997; Haveman et al., 1999; Hallbeck and Pedersen, 2012). Our aim is to understand how communities in fractured granite form and what these findings mean in a broader context of a study site.

Patterns of phylogenetic relatedness within a community enable detection of selection as a processes governing community assembly (Horner-Devine and Bohannan, 2006; Emerson and Gillespie, 2008). Beta diversity is a measure of differences in taxa identities, abundances and phylogenies among locations within a region of interest (Graham and Fine, 2008; Cavender-Bares et al., 2009; Anderson et al., 2011). This measure represents the variation among communities, linking the local community (alpha diversity) to other communities within the region (gamma diversity). Because both selection and dispersal (amongst other neutral processes) contribute to beta diversity (Vellend, 2010), these measures can help explain the organization and functioning of microbial communities within a region of fractured granite. Understanding how a pool of taxa assemble and are maintained over a region will inform how active *in situ* metabolic processes evolve in space and time, how biogeochemical processes differ across a formation and ultimately how functional and phylogenetic diversity (PD) can affect the solubility and transport of compounds through the formation.

Recently, a conceptual ecological framework (Vellend, 2010) was transformed into an operational framework and applied to a meta-community to compare the relative influences of deterministic and neutral processes on subsurface microbial communities within and across the Ringold and Hanford geologic formations (Stegen et al., 2015). Under this framework, meta-community dynamics differentiate into a combination of selection, dispersion and random processes; differentiation is through the combined results of the beta diversity metrics for the β-Nearest Taxon Index (βNTI) and the Raup Crick index (Chase et al., 2011). βNTI is a measure of a community’s phylogenetic composition in terms of relatedness of co-occurring taxa relative to a meta-community (Webb et al., 2008). These calculations, therefore, provide a measure of the phylogenetic relatedness within and between each sampling location. Raup-Crick beta diversity is a measure of dissimilarity between communities compared to a null expectation. The resulting Raup-Crick beta diversity, relative to the corresponding alpha and gamma diversity, provides an indication of whether deterministic or neutral processes have influenced overall community dynamics. It may be that no process dominates and the communities are randomly assembled (Stegen et al., 2015).

In this study, we evaluated a bacteria meta-community of 16S rRNA gene libraries from multiple groundwater-filled granite fractures located within the boundary of Chalk River Laboratories, Ontario, Canada. We accessed groundwater to a depth of 670 m from drill holes transecting low permeability stacked gneiss assemblages underlying the study site. We compared the alpha and beta diversity to the measured groundwater variables and to the spatial locations of the sampled fractures. By comparing different sampling locations, we were able to relate ecological assembly processes to possible environmental and spatial drivers that govern the structure of the microbial communities.

## Materials and Methods

### Sampling Groundwater from Fracture Zones

**FIGURE 1:**
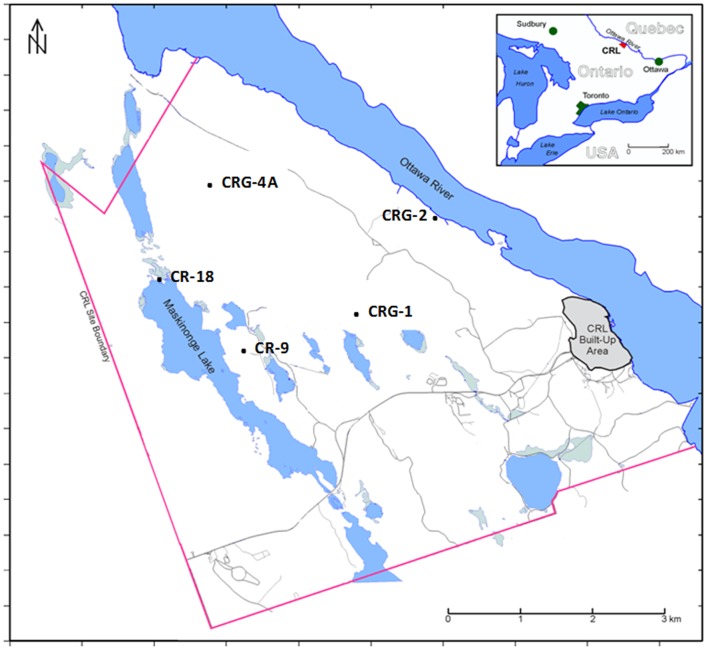
**Map of the Chalk River Laboratories site located in Eastern Ontario, Canada showing the approximate locations of boreholes included in the study**.

The locations of boreholes within the study site are shown in **Figure 1**. The Mattawa fault (Ottawa River) and the Maskinonge Lake fault, also shown in **Figure 1**, bound the study site. Diabase dykes traversing the site form another boundary. These features may isolate groundwater into different zones across the area of study. Underlying the site are gneiss forming stacked assemblages consisting of an overlying and underlying garnet-poor assemblage, and a central garnet-rich assemblage. A more detailed description of the site is provided in the Supplementary Information. Samples were collected for geochemical and microbiological analysis from four sealed boreholes (Westbay^TM^ Multilevel Groundwater Monitoring System (Schlumberger Water Services)) and one open borehole.

**FIGURE 2:**
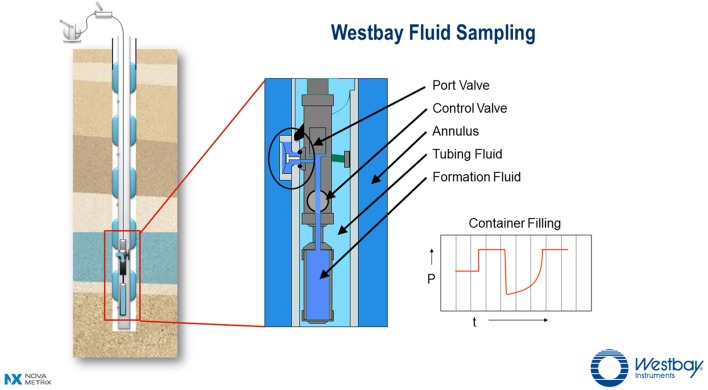
**Schematic of the Westbay System for multi-level groundwater monitoring (used with permission of Nova Metrix Ground Monitoring Ltd)**.

**Figure 2** shows a schematic of a borehole with an installed Westbay System^®^ for multi-level groundwater monitoring. This Figure illustrates how the Westbay tubing and packers isolate multiple zones within the borehole thus preventing unnatural vertical groundwater flow within the borehole itself. The tubing fluid is isolated from the formation fluid. In this arrangement ambient formation fluid flow can pass through the annulus. From inside the tubing, formation fluid can be accessed by lowering a Westbay sampler and container assembly (also shown) to normally closed valved ports positioned between the packers. A larger schematic illustrates a deployed Westbay sampler assembly that is engaged at a selected port. Once the sampler is positioned and engaged, the remotely operated control valve in the sampler is opened to allow formation fluid from the zone to flow into the empty container. The process is monitored by observing changes in fluid pressure during the sequence of operations (see a typical trace of pressure vs. time in **Figure 2**). Once the container is filled, the sampler valve is closed to seal the formation fluid inside the container at *in situ* pressure. The assembly is disengaged from the port (the port valve automatically closes at this time) and the fluid in the sealed container is retrieved to the surface for further handling.

We sampled three fracture locations isolated by borehole CR-9 and seven fracture locations isolated by boreholes CRG-1, CRG-2, and CRG-4A (**Table 1**). The open borehole, CR-18, was accessed by lowering a pump line into the borehole. The groundwater sampling tube assembly consists of four 250 mL stainless steel tubes connected in series by tubing and swagelok fittings. Prior to each sampling, the tubes were sterilized by autoclave and the fittings were sterilized by washing them with 70% ethanol. Validation of the sterilization and transport procedures was performed using sterilized water and PCR with bacterial rRNA 16S primers (Muyzer et al., 1993). Since the tube assemblies contacted only the interior of the casing surface, the probability of introducing surface microbes into the sampled volumes was minimal.

**Table 1:** Geochemistry and descriptive parameters for groundwater from each borehole sampling location.

		CRG-1				CRG-2		CRG-4A		CR-9		CR-18
							
Interval:		3	6	8	14	2	4	9	2	5	12	–
Sampling Date m/d/y		05/30/2011	05/31/2011	06/01/2011	06/02/2011	09/20/2011	09/21/2011	08/23/2011	11/04/2008	11/06/2008	11/13/2008	09/22/2011
Easting^∗^	m	313743.6	313760.8	313771.9	313815.2	314757.5	314777	312203.4	312721.5	312449.75	312416.14	311262.7
Northing^∗^	m	5102838	5102857	5102870	5102917	5104142	5104160	5104452	5102262	5102130.9	5102120	5103333
Depth	m	145	244	307	532	72	126	604	96	279	672	5
Elevation	m^∗∗^	31	-68	-131	-356	92	38	-440	29	-154	-426	111
Conductivity	μs/cm	233	292	220	302	223	210	290	258	317	4225	213
Temperature	°C	9.0	8.6	9.1	11.1	9.6	8.4	11.7	8.6	8.4	11.7	-
pH		8.5	8.5	8.6	9.1	8.6	9.4	7.9	9.3	9.1	8.5	8.5
DOC	mg/L	25.9	23.5	23.2	7.9	23	17.4	18.4	22	19	1.9	1
HCO_3_^-^	mg/L	146.5	131.1	130.7	76.6	116.8	83.1	124.3	121.9	91.5	19.8	79.5
Cl^-^	mg/L	5.4	31	6	41	0.5	1	14	58	28	1,572	91
SO_4_^-2^	mg/L	1.3	0.5	0.1	0.2	11.4	10.8	2	7.9	7.3	83	25
NO_3_^-^	mg/L	<mdl	<mdl	<mdl	<mdl	<mdl	<mdl	<mdl	<mdl	<mdl	<mdl	<mdl
PO_4_^-3^	mg/L	<mdl	<mdl	<mdl	<mdl	<mdl	<mdl	<mdl	<mdl	<mdl	<mdl	<mdl
Ba^+2^	mg/L	0.02	0.04	0.02	0.01	<mdl	<mdl	0.01	0.01	0.01	0.14	0.1
Ca^+2^	mg/L	10.5	22.9	10.8	4.2	11.2	3.1	13	9	7.8	325	18.1
Mg^+2^	mg/L	1.5	4.1	1.3	0.4	1.2	0.2	1.2	0.8	0.3	8.1	1.1
Na^+^	mg/L	40	31	36	61	39	43	53	47	64	510	80
Fesoluble	μg/L	40	60	80	20	20	100	50	80	30	<mdl	50
Mnsoluble	μg/L	30	30	20	10	10	<mdl	20	10	<mdl	80	10


*na, not available; mdl, method detection limit. ^∗^Universal Transverse Mercator coordinates, zone 18. ^∗∗^Elevation relative to sea level.*

After sampling and transport to the laboratory, opening the filled tubes and dispensing the sampled groundwater, took place inside of a glove box under an atmosphere of filtered nitrogen gas. Inside the glovebox, the groundwater pH (Beckman PHI 265 pH/Temp/mV meter (Beckman Coulter, Inc.)) and conductivity (YSI Model 30 Conductivity Meter (YSI Inc., Yellow Springs, OH, USA) were measured and aliquots for elemental analysis were filtered through a 0.45 μm filter (isopore polycarbonate, Millipore, Billerica, MA, USA) and immediately acidified using nitric acid (ultra-trace grade, Seastar^TM^, Baseline^®^, Fisher Scientific, Ottawa, ON, Canada). Elemental composition of the groundwater was determined by inductively coupled plasma-mass spectrometry (ICP-MS, using either a Varian 820-MS (Agilent Technologies, Inc.) or an Element XR (Thermo Scientific)) and by inductively coupled plasma atomic emission spectroscopy (ICP-AES, Optima 3300, Perkin Elmer). Anion concentrations were determined using a Dionex 3000 ICS ion chromatograph (Dionex, Sunnyvale, CA, USA). Dissolved organic (DOC) and inorganic carbon (DIC) were determined using a Dohrmann, model Phoenix 8000-UV Persulfate TOC Analyzer (Teledyne Teckmar, Mason, OH, USA). The excitation/emission of fluorescein in the groundwater provided a measure of residual drill water.

### Characterization of the Bacterial Assemblages

#### Direct Cell Counts

Epifluorescent direct cell counting was used to enumerate total microbes in the groundwater. Triplicate 1 mL volumes of the groundwater was incubated with a DNA intercalating dye then filtered onto black polycarbonate filters (0.22 μm, Millipore) for viewing and counting.

#### Nucleic Acid Extraction, Quantitative PCR, and Creation of 16S rRNA Gene Libraries

Biomass from groundwater samples (0.8–1.0 L) was collected through filtration onto a sterile 47 mm diameter polyethersulfone filter with a 0.22 μm nominal pore size, using a sterile 100 mL filter housing (Millipore Corp., Billerica, MA, USA) within 24 h of sample collection. Nucleic acids were extracted from the filters immediately, using the UltraClean^®^ microbial DNA isolation kit (Mo Bio Laboratories, Inc) (CRG-1, CRG-2, CRG-4A, CR-18) and the Rapid Water^®^ DNA Isolation Kit (Mo Bio Laboratories, Inc) (CR-9).

Quantitative PCR (qPCR) was used to enumerate the number of copies of the bacterial ssu rRNA genes as a proxy for population density (copies/mL). The group-specific primers used for qPCR were 27F and 338R for bacteria (250 and 125 nM, respectively) and A8F and A44R (500 nM each) for archaea (Nakatsu and Marsh, 2007). Each qPCR contained 2 μL of sample DNA, primers, and 15 μL of 2x SYBR^®^ Green PCR Master Mix (Applied Biosystems), with a total volume of 30 μL. Each experimental DNA was amplified in triplicate. The nearly full-length ssu rRNA gene from the bacterium *Thermacetogenium phaeum* or archaeon *Methanospirillum hungatei* JF-1, cloned into the pCR4 vector (TOPO TA Cloning, Invitrogen), were used for triplicate control reactions. Ten-fold serial dilutions of the control DNA were used for the standard curve, ranging from 3.42 × 10^3^ to 3.42 × 10^9^ copies per reaction. Thermal cycling and detection was performed with a 7300 Real Time PCR System (Applied Biosystems). The cycling conditions for bacterial qPCR included 10 min at 95°C followed by 30 cycles of 30 s at 96°C, 45 s at 55°C, and 45 s at 72°C. The cycling conditions for archaeal qPCR were identical, with the exception of 40 cycles of amplification. The amplified product from each reaction was visualized on an agarose gel (1% w/v in 0.5x TAE, 80 v, 1 h) stained with ethidium bromide to confirm the product size and lack of primer dimer.

The 16S rRNA gene libraries were prepared directly from bacterial qPCR products by pooling and purifying triplicate reactions using the Wizard PCR Preps DNA Purification System (Promega, Madison, WI, USA). The negative qPCR control reactions (no DNA added) were also included. Triplicate PCRs for each library were pooled and purified as described above, and a unique barcode (forward primer only) was attached with the primers TiA-8nt-M13F and TiB-M13R, along with the necessary 454 Fusion A and B tags (454 Life Sciences, Branford, CT, USA). A customized 2-step barcoding approach was used similar to that used by Herbold et al. (2015). Purified libraries were then barcoded with a unique 8 bp barcode, which consisted of a PCR with 5 μL of template, 1X PCR buffer, 0.625 U Taq DNA polymerase (Fermentas, Thermo Fisher, Waltham, MA, USA), and 0.05 μM of the primers (TiA-8nt-27F and TiB-338R) in a total volume of 25 μL. Six cycles of PCR were carried out; otherwise, reaction conditions were identical for bacterial qPCR. The tagged PCR products were purified again using the Wizard PCR Preps DNA Purification System and quantified using the Qubit HS assay (Life Technologies, Carlsbad, CA, USA). Equimolar amounts of each library were pooled and then sequenced using a Genome Sequencer FLX instrument with the GS FLX Titanium series reagents (454 Life Sciences).

#### Analysis of 16S rRNA Gene Libraries

Raw sequence data was quality filtered and demultiplexed using QIIME (Caporaso et al., 2010b). Demultiplexed libraries were denoised to reduce the error profile inherent within 454 pyrosequencing (Reeder and Knight, 2010).

Sequences were clustered into operational taxonomic units (OTUs) at 97% similarity with USEARCH (Edgar, 2010), and chimeric sequences were removed using *de novo* and reference-based searches with UCHIME (Edgar et al., 2011). Representative sequences for each OTU were aligned using PyNAST against the SILVA reference database [release 123, (Caporaso et al., 2010a; Quast et al., 2013)] and classified using the RDP naïve Bayesian classifier with the SILVA database (release 123).

##### Material Availability

The 16S rRNA sequence reads were submitted to the short read archive under accession number SRR1261803.

### Selection, Dispersal, and Random Processes

#### Phylogenetic Measures of Assemblage Diversity

The R package (R Core Team, 2015), ‘picante’ (Kembel et al., 2010) was used to calculate taxa richness, PD, mean pair wise distance (MPD), and the mean nearest taxon distance (MNTD). These calculations compare taxa within assemblages from each sampling location with the taxa from the meta-community tree. PD is the summed phylogenetic branch length connecting all taxa in the meta-community (Faith, 1992). Taxa richness was also derived from the PD calculation by scaling the maximum edge length to one, the resulting PD is the taxa richness. To account for differences in taxa richness on PD, we evaluated PD by using the standardized effect size for PD (by the function ses.pd, abundance.weighted = TRUE). The null model ‘taxa.labels’ was set at 999 randomizations.

The MPD and MNTD calculations provide measures of co-existence and phylogenetic turnover between the assemblages that make up the meta-community. MPD compares taxa relatedness to the average tree edge length while MNTD compares taxa relatedness at closer phylogenetic depth. To calculate MPD and MNTD, a pair-wise distance matrix of the meta-community tree was first calculated using the function cophenetic(); the assemblages from each sampling location were compared with the mean of the non-diagonal elements (MPD) and with the smallest non-diagonal matrix values for each taxon (MNTD). Patterns of co-existence – of closely related (clustered) or more distantly related (overdispersed) taxa – were evaluated using the standardized effect size for MPD (by the function ses.mpd, abundance.weighted = TRUE) and for MNTD (by the function ses.mntd, abundance.weighted = TRUE). For each of these calculations the model ‘taxa.labels’ was set at 999 randomizations.

#### Beta Diversity

To evaluate the strength of selection, phylogenetic turnover between communities was calculated as the β-nearest taxon index (βNTI) (Stegen et al., 2013). These calculations were performed within the R environment using the codes provided by Stegen et al. (2013) and that are available from Github at https://github.com/stegen/Stegen_etal_ISME_2013/blob/master/bNTI_Local_Machine.r. Within this code, the standardized effect size for MNTD between sampling locations, βMNTD (abundance.weighted = TRUE) is calculated and then multiplied by minus one to give values for the β-nearest taxon index, βNTI.

For those pair wise comparisons that were not significant for selection by βNTI, the strength of dispersal on assembly was evaluated by calculating Raup-Crick (Chase and Myers, 2011; Stegen et al., 2013) that was extended to include comparisons between relative abundances. These calculations helped to discern between the contributions of limiting and homogenizing dispersal. These calculations were performed within the R environment using the codes provided by Stegen et al. (2013); these are available from Github at https://github.com/stegen/Stegen_etal_ISME_2013/blob/master/Raup_Crick_Abundance.r. The result of this analysis is referred to as RC_bray_. The code was run at 999 repetitions to generate a distribution of null values.

#### Distance Decay of Similarity

The distance decay of similarity was determined using Bray–Curtis similarity (from the R package ‘picante’) and weighted UniFrac similarity (from the R package ‘GUniFrac’). The geographic distance between sampling locations was calculated using the function earth.dist from the R package ‘fossil’ and the data fit using the function scatter.smooth().

### Spatial Descriptors and Selection of Explanatory Variables

A Moran’s eigenvector map (MEM), that was created by principle coordinates of neighbor matrices (Borcard and Legendre, 2002; Legendre et al., 2009) from within the R packages ‘sdep’ and ‘adespatial,’ was used to build a matrix of spatial eigenvectors from a distance matrix of Easting and Northing, zone 18, Universal Transverse Mercator coordinates for each borehole interval (**Table 1**). The functions used to create the spatial weightings matrix were nbtri(), that converts the spatial coordinates of the sampling locations into a distance neighbors map, and the function nb2listw() that creates the weightings matrix from the neighbors map. The eigenvectors with positive Moran’s I values reveal different spatial structures over the entire range of scales encompassed by the geographical sampling area. The first MEM values generated in the analyses represent broader spatial structures, and the last MEM values represent finer spatial structures. The MEM values were combined and standardized with the geochemistry for dissolved organic carbon, bicarbonate, sulfate, iron, manganese, and chloride (**Table 1**) using the function decostand() (within the package ‘vegan’). The standardized values were then used to perform a constrained redundancy analysis (RDA). Standardized taxa abundances (by the function decostand() within the package ‘vegan’, specifying the Hellinger transformation) was the dependent variable. The functions ordistep() and ordiR2step() were then used in forward model selection to define the overall adjusted *R*^2^ value.

## Results

### Groundwater Geochemistry

**Table 1** shows the groundwater concentrations of dissolved organic carbon, bicarbonate, sulfate, iron, manganese and major ions from the isolated fractures. The groundwater from fractures isolated by boreholes CR-9 and CR-18 displayed higher chloride and sulfate concentrations than the groundwater from boreholes CRG-1, CRG-2, and CRG-4A. The bicarbonate content of the groundwater decreased with borehole interval depth and displayed a high of 146.5 mg/L (borehole CRG-1) and a low of 19.8 mg/L (borehole CR-9). The soluble iron content of the groundwater tended to increase with depth for groundwater taken from the CRG-boreholes, ranging from a low of 20 μg/L to a high of 100 μg/L, and tended to increase with depth for groundwater taken from borehole CR-9, ranging from a high of 80 μg/L to being below detection. The soluble manganese content of the groundwater tended to decrease with depth in groundwater taken from CRG-boreholes, ranging from a high of 30 μg/L to a being below detection, and to tended to increase with depth in groundwater taken from borehole CR-9, ranging from below detection to 80 μg/L. The nitrate and phosphate ions were below detection across all samples. A principle component analysis of the major ions and stable oxygen isotope (**Supplementary Figure S1**) suggested that the groundwater is of meteoric origin. The groundwater pH was slightly alkaline ranging from pH 7.9 to 9.4 and the *in situ* temperature ranged from 8.8 to 11.7°C.

### Bacterial Composition of the Groundwater

Bacteria were dominant in the groundwater, relative to the Archaea. Estimates of the 16S rRNA gene copy numbers for Archaea were at or below the values of the negative control (0 to <100 cells/mL; data not shown), whereas the estimates of bacterial 16S rRNA genes were between 7.8 × 10^4^ and 1.3 × 10^7^ copies per mL of groundwater filtered (**Supplementary Table S1**). Direct counts of cells by DNA staining estimated the cell densities to be between 5.2 × 10^4^ and 1.1 × 10^6^ cells/mL (**Supplementary Table S1**).

The relative abundance of all other OTUs (<1% on average) is grouped as “Other OTUs.” with their collective relative abundance (%) denoted in each box. The dendrogram above the samples depicts similarity among the microbial communities based on unweighted UniFrac distances.

**FIGURE 3:**
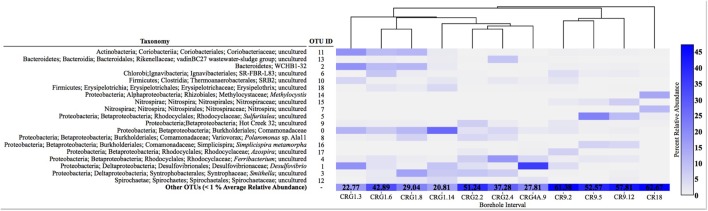
**Diagram showing the taxonomic identification of the most abundant operational taxonomic units (OTUs, >1% average relative abundance) and their relative abundance in each sample (heatmap).** The relative abundance of all other OTUs (<1% on average) is grouped as “Other OTUs.” with their collective relative abundance (%) denoted in each box. The dendrogram above the samples depicts similarity among the microbial communities based on unweighted UniFrac distances.

A total of 114,787 sequences were grouped into 780 OTUs; these varied in their distribution across all libraries (**Supplementary Table S2**). Most of the OTUs were low abundance across the meta-community as only 19 of all the OTUs were represented >1% of the average relative abundance. The distribution of these abundant OTUs is presented in a heatmap (**Figure 3**). The most abundant OTUs identified were a Comamonadaceae (OTU 0), a *Desulfovibrio* (OTU 1) and a Bacteroidetes WCHB1-32 (OTU 2). The other abundant OTUs were uncultured representatives of Betaproteobacteria [including an *Azospira* (OTU 17), *Sulfuritalea* (OTU 5) and a *Ferribacterium* (OTU 4) from the Rhodocyclales and *Simplicispira* (OTU 16) and *Polarimonas* (OTU 8) from the Burkholderiales; Deltaproteobacteria, Bacteroidetes, Firmicutes, Alphaproteobacteria, and Nitrospirae. The heatmap in **Figure 3** also shows the proportion of OTUs making up <1% of the average relative abundance that were combined together as “other OTUs.” The proportion of low abundance OTUs were highest from assemblages within borehole CR-9 (52–63%) and lowest from assemblages within borehole CRG-1 (22–43%).

When viewed at a higher taxonomic level, the most abundant OTUs (**Supplementary Table S2**) were members of the class Betaproteobacteria (20–40%). The other abundant OTUs (**Supplementary Table S2**) were members of the Deltaproteobacteria (1–49%), Bacteroidetes (3–26%), Alphaproteobacteria (1–19.8%) and Firmicutes (<1–16%). The assemblages from within the fracture zone accessed by borehole CR-18 had the highest abundances of Acidobacteria (12.6%) and Alphaproteobacteria (19.8%). Abundant *Nitrospiracaea* (up to 21.5%) were found in groundwater from boreholes CR-9 and CR-18. Notably, 49% of the bacteria community within the fracture zone accessed by borehole CRG-4A was composed of a single taxon, OTU 1 (shown in the heatmap in **Figure 3** and identified as *Desulfovibrio*; this OTU was also found in the assemblages from boreholes CRG-1 (intervals 3, 6, and 14), CRG-2 (interval 2) and in CR-9 (interval 2).

### Influences of Selection and Dispersal

#### Diversity and Co-existence

**FIGURE 4:**
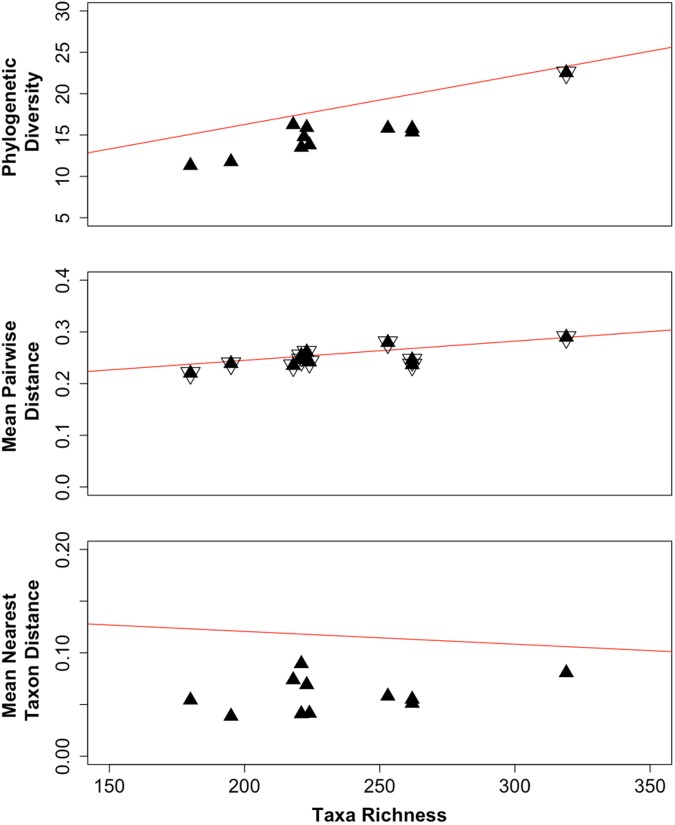
**Observed values (black triangles) for phylogenetic diversity (PD, top panel), mean pairwise distance (MPD, middle panel) and mean nearest taxon distance (MNTD, bottom panel) of assemblages relative to the taxa richness.** The regression line is shown for the corresponding null mean values (red lines). Assemblages marked with an open triangle indicate the observed values were within two standard deviations of the null mean values.

By normalizing the difference between observed values and the null mean as multiples of the standard deviation significant differences from the null expectation are those values (*z*-values) that are two or more standard deviations from the mean (Vellend et al., 2010; Cadotte and Davies, 2016). The observed values for PD, MPD, and MNTD are plotted in **Figure 4** (black filled triangles) as these measures relate to the observed taxa richness (the calculated values are listed in **Supplementary Tables S3**–**S5**, for PD, MPD and MNTD, respectively.) The regression lines for the corresponding null expectations are shown in red. When the values for an assemblage (sampling locations) displayed observed PD, MPD, or MNTD values that were within the respective null expectations for the meta-community, these locations are marked in **Figure 4** with both a filled and an open triangle. Otherwise, the values were outside the null expectation and were depicted with a single black triangle; these assemblages display either clustering (located below the regression line for the null expectation) or over dispersion (located above the regression line for the null expectation). We found that at the phylogenetic depth for MPD (**Figure 4**, middle panel), the observed MPD values for all the assemblages were within null distribution, which indicates the assemblages were dispersed evenly relative to the meta-community. Only one observed PD value (**Figure 4**, top panel) was within two standard deviations of the null mean values (marked with both open and closed triangles); this assemblage corresponds to borehole CR-9, interval 2 that had the highest taxa richness (**Supplementary Table S3**). The remainder of the observed PD values, and all of the observed MNTD values (**Figure 4**, bottom panel), were more than two standard deviations from the corresponding null expectations. Since these values were below the regression line for the null expectation, the assemblages from these locations displayed lower taxa richness (PD) and co-occurrence of phylogenetically related taxa (MNTD). These measures indicate selection as a driver shaping each of these communities.

#### Beta Diversity

To help identify the basis for the lower assemblage level diversity and the co-occurrence of related taxa, beta diversity was also calculated and interpreted according to the ecological modeling framework of Stegen (Stegen et al., 2015) for selection, dispersal and drift. A listing of the null comparisons for βNTI and Raup-Crick (as RC_bray_) are provided in **Table 2**. Briefly, phylogenetic beta diversity βNTI values that are more than two standard deviations on either side of the mean (values of +2 or higher, and of -2 or lower) indicate selection as a driver for community assembly. If a βNTI value falls within the null expectation, namely between -2 and +2, observed differences between the paired sampling locations are random. The resulting distance matrix for βNTI is listed in **Supplementary Table S6**. Values outside the null expectation for βNTI occur between sampling locations within boreholes CR-9 and CR-18 when paired with each other and the other sampling locations. The phylogenetic beta diversity, therefore, indicates selection as one of the drivers of the community assembly.

**Table 2:** Beta diversity relationship to ecological process (Stegen et al., 2013).

Ecological process:	βNTI	RC_bray_
Selection (variable)	> +2	–
Selection (homogeneous)	< -2	–
Dispersal (limiting), drift	Null	> +0.95
Dispersal (homogenizing)	Null	< -0.95
Random assembly	Null	null


To discern the contribution of dispersal on assembly, the sampling locations with βNTI values that were within the null expectation were therefore also used to calculate beta diversity based on taxa identities, Raup-Crick. The Raup-Crick values were further processed using Bray–Curtis dissimilarity, therefore, for RC_bray_ values that were more than +0.95 or less than -0.95 corresponded to values that were more than two standard deviations on either side of the mean.

When values for βNTI are also within the null range and RC_bray_ values are outside of the null range, then either limiting dispersal or homogenizing dispersal, as listed in **Table 2**, are drivers of community assembly, respectively. The resulting distance matrix for RC_bray_ is listed in **Supplementary Table S7**. When the observed values for both βNTI and RC_bray_ fall within their null distributions, the bacterial dynamics between paired sampling locations are random. Based on the combinations of βNTI and RC_bray_ values from **Supplementary Tables S6** and **S7**, the assemblages from boreholes CRG-1, CRG-2, and CRG-4A indicate limiting dispersal and random processes. The phylogenetic beta diversity, therefore, identifies dispersal and random processes as drivers of the community assembly.

#### Distance Decay of Similarity

**FIGURE 5:**
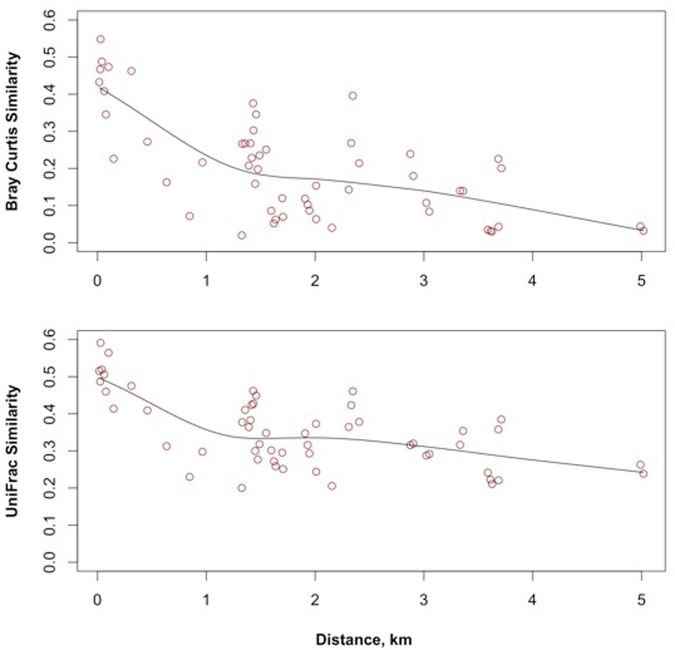
**Bray–Curtis and UniFrac distance decay relationships of groundwater bacteria across the study site**.

Distance decay refers to the decrease of similarity as the distance between observations increases. A negative relationship between distance and similarity is implicit in taxa turnover occurring along an environmental gradient but can also occur by isolation created by geography (Nekola and White, 1999). When the decay in similarity occurs along an environmental gradient, the cause of the decay is attributable to competition and fitness, or environmental filtering. When the decay in similarity occurs by spatial isolation, the combined influences of space and time limit movement across landscapes (Nekola and White, 1999). The decay of Bray–Curtis and weighted UniFrac similarities of the meta-community is shown in **Figure 5**. Both similarity measures display a rapid decay distance between 0 and 1.5 km and a shallower decay distance between 1.5 and 5 km.

### Identification of Significant Environmental and Spatial Variables

Next, we compared taxa abundances with the geochemical and spatial variables by performing a constrained RDA followed by forward selection to define a reduced model. The RDA indicated whether there were significant relationships but did not identify explanatory variables. The resulting global adjusted *R*^2^ was used as an upper limit for subsequent comparisons to identify the explanatory variables. The variables included were the prospective electron donor and electron acceptor compounds from the groundwater: dissolved organic carbon, bicarbonate, sulfate, iron, and manganese (**Table 1**), and the signatures for a proposed seawater origin (**Table 1**, **Supplementary Figure S1**). The spatial variables were from the Moran’s eigenvector map (MEM) analysis. The details of the modeling are provided in the Supplementary Information.

**FIGURE 6:**
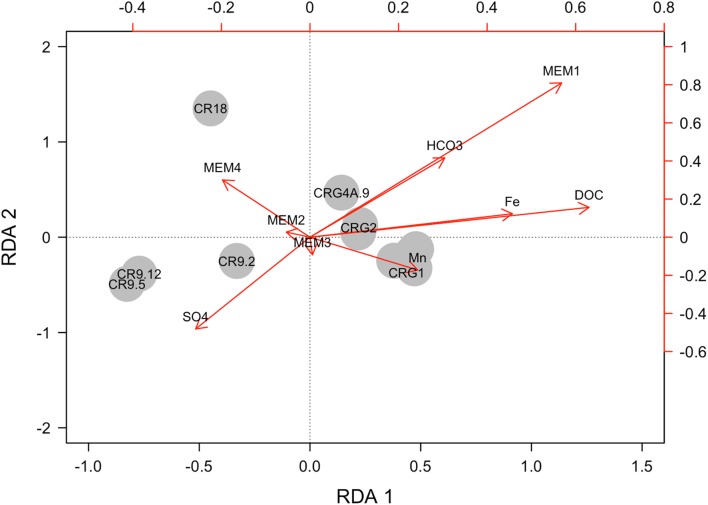
**Explanatory power of selected environmental and spatial variables on taxa abundances.** The axes are the first and second coefficients from the redundancy analysis (RDA). Global adjusted *R*^2^ of 29.4%. The sampling locations are labeled according to the borehole and interval used to access the groundwater.

The resulting model for taxa abundances accounted for 29.4% of the global adjusted *R*^2^ variance, *p* < 0.01. The contributions of the explanatory variables for each sampling location are shown in **Figure 6**. There is clear separation between boreholes and the accessed intervals. Also shown in **Figure 6** are the vectors for environmental variables: organic and inorganic carbon, iron, manganese, and sulfate, and the first four spatial variables: MEM1, MEM2, MEM3, and MEM4. Initial model selection identified the two largest spatial scales, MEM1 and MEM2 (*p* < 0.05) as the variables associated with taxa abundances. On model reduction, MEM1 was identified as being the most significant variable accounting for 20.4% of the adjusted *R*^2^ variance.

**FIGURE 7:**
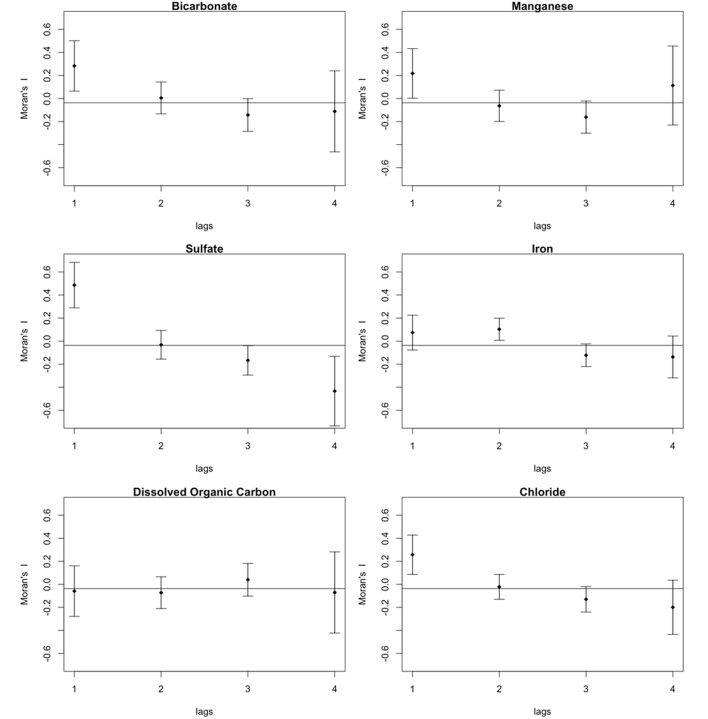
**Moran’s I correlogram showing spatial correlation of some major groundwater components; bicarbonate, manganese, sulfate, chloride, and iron, but not for dissolved organic carbon**.

None of the geochemistry in the model was found to be significant. Spatial correlograms support these model outcomes – bicarbonate, iron, manganese, and sulfate each display spatial correlation (**Figure 7**). Other components of the groundwater, the dissolved organic carbon, chloride and pH were not spatially correlated (**Figure 7**). The groundwater meta-community appears to be subject to dispersal limitation. Relative to the meta-community, therefore, individual assemblages appear to be subject to random variation in population and size caused by drift.

## Discussion

The microorganisms found in terrestrial subsurface environments are abundant, accounting for 2–19% of Earth’s biomass (Kallmeyer et al., 2012) and contribute to important biogeochemical cycles, but their relative inaccessibility means that they are largely unknown. The terrestrial subsurface environment potentially represents a unique script for ecological function, as physical separation likely limits dispersion, which is highly dependent upon the flow of groundwater through a formation. Rock–water interactions mean that groundwater within fracture zones that traverse rock layers can transition to different geochemistry (Swanner and Templeton, 2011). This change, in turn, can affect the composition and diversity of local microbial assemblages, which can vary considerably across short distances (Pedersen, 1997; Gihring et al., 2007; Sahl et al., 2008). Furthermore, taxa richness in the subsurface tends to decrease with increasing depth, and those few taxa present at greater depths often represent novel phylogenetic lineages (Gihring et al., 2007; Sahl et al., 2008). The physical separation of these low abundance microbial assemblages may explain their apparent evolutionary divergence and diversification (Papke and Ward, 2004). We posit that the microbial assemblages within the terrestrial subsurface provide additional evidence that microbial diversity follows an apparent pattern of biogeography on scales of a few millimeters to thousands of kilometers (Martiny et al., 2006), challenging the once long-standing assumption of unlimited dispersal of microorganisms that are selected by the environment alone. These patterns in microbial diversity are likely driven by the combined influences of selection, dispersion, and drift (Hanson et al., 2012). Whereas, the dynamics of the uncultivated microbial diversity over space and time can reveal patterns of energy flow and biogeochemical processes within the terrestrial subsurface.

The region under study at Chalk River Laboratories (located in Ontario, Canada and shown **Figure 1**) is underlain by granite that is fractured to depths of several hundreds of meters. In this Shield environment, these fractures provide the pathways for local and regional groundwater flow. Dispersal limitation of groundwater microbes within these fractures would be expected to represent a virtual time capsule of extant microbes, relative to a historical regional pool of taxa continuously being transported to these fractures via recharge. This phenomenon would date back to the period of glacial retreat approximately 11,000 years before present; with an exception that, once isolated within these fractures, ongoing dynamic processes of selection, dispersal and drift continued to influence the community assembly.

Bacterial communities were characterized from groundwater obtained from multiple discrete fractures isolated by five boreholes drilled into the rock mass underlying the study site (**Figure 1**). Determinations of biomass and taxa distributions were characterized in order to gain insight on likely metabolic reactions prevalent at each sampling location. We also analyzed the meta-community within an ecological framework by calculating bacterial diversity at the local and regional scale. The resulting diversity, within and between assemblages, provided insight on the relative contributions of selection, dispersal and drift. The contributions of possible geochemical gradients and distance on the meta-community was also evaluated by three approaches: distance decay of similarity; RDA; and by calculating Moran’s I.

The results represent an improved understanding of saturated fractured granite systems. The microbial community density in our samples was between 10^4^ and 10^5^ cells/mL, which is in line with densities between 10^3^ and 10^7^ cells/mL reported for other terrestrial granitic subsurface environments (Pedersen, 1997). Unlike other granitic groundwater systems; however, methanogenic archaea were not a significant proportion of these communities since Archaea were below detection based on qPCR (**Supplementary Table S1**).

The bacterial assemblages across boreholes and depths were composed of many, mainly low abundance (<1% relative abundance) taxa or OTUs. This abundance distribution pattern is commonly seen in microbial ecology (Nemergut et al., 2013) in which only a few taxa are present in abundance; the majority of the taxa present are rare. Higher phylogenetic/taxonomic groups (phyla, classes) were shared across sites, consisting mainly of members of the phenotypically diverse Betaproteobacterial families Comamonadaceae (Willems, 2014) and Rhodocyclaceae (Oren, 2014). The OTUs detected across the boreholes were most closely related to uncultivated taxa; however, the closest cultivated representatives are capable of metabolism as diverse as oxic, microoxic, and anoxic growth through chemoorganoheterotrophy, oxidation of iron, sulfur, and hydrogen, and the reduction of nitrate, iron and manganese, and even N-fixation. Members of the Deltaproteobacteria families Desulfovibrionaceae and Synthrophaceae were also present in relative abundance, suggesting sulfate reduction and syntrophy in anoxia as probable metabolic niches across sampling locations. Finally, two OTUs related to members of the Nitrospiraceae were abundant in CR-18 and CR-9 with increasing depth. These chemolithoautotrophs are capable of nitrite oxidation (*Nitrospira*) and iron oxidation (*Leptospirillum;* Lücker et al., 2010), or anaerobic hydrogenotrophic sulfate reduction (*Thermodesulfovibrio*) (Daims, 2014). *Nitrospira* were shown to play a key role in the cycling of nitrogen in a deep terrestrial granitic system in Henderson, CO, USA (Swanner and Templeton, 2011).

By evaluating the study site as individual sampling locations and as a meta-community across all sampling locations, we were able to detect probable drivers of the meta-community assembly. Within each sampling location we detected assemblages with lower PD compared to the meta-community (**Figure 4**, top panel). Values for PD change in proportion to the taxa richness (Cadotte and Davies, 2016). Fewer taxa within an assemblage (sampling location) than what was is represented by the meta-community, therefore, would be reflected as lower PD. Lower than predicted PD was also found in assemblages of suspended marine bacteria (Horner-Devine and Bohannan, 2006) and of soil bacteria (Goberna et al., 2014). Within each community we also found that co-occurrence of these fewer taxa spanned the meta-community phylogeny at the phylogenetic depth of the mean pairwise distances (MPD, **Figure 4**, middle panel), signifying even dispersion. However, their co-occurrence at the phylogenetic depth of the MNTD (**Figure 4**, bottom panel), reveals they are more closely related than predicted by the meta-community, signifying clustering. These outcomes support selection as a driver of the local subsurface bacterial assembly.

One possible selective force is environmental filtering. This filtering refers to the incompatibility of some taxa to environmental factors, and so is thought to explain the co-occurrence of phylogenetically related taxa that share evolutionarily conserved traits, while more distant taxa, lacking similar traits, fail to become established (reviewed in Mayfield and Levine, 2010). Co-occurrence of related taxa within an assemblage is considered unlikely under the concept of competition and exclusion (Webb et al., 2002). We have, in our analysis, fewer taxa based on PD, dispersion at more distant phylogenetic depth (MPD) and clustering at more related phylogenetic depth. Mayfield and Levine (Mayfield and Levine, 2010) recently proposed that competition can explain both dispersion and clustering without invoking environmental filtering as a separate mechanism. Instead, either dispersion (niche) or clustering (competitive ability) may be favored, depending on the relative strengths of phylogenetically correlated niche differences and the competitive ability of shared traits. Similarly, niche differences unrelated to phylogeny, when combined with competitive ability that relates to phylogeny, can also explain clustering. For example, sulfate reduction is not a phylogenetically distinct trait, since this trait is shared between some members of the divergent lineages Deltaproteobacteria and Firmicutes. Abundant members from these lineages were observed in the study described here (**Figure 3**; **Supplementary Table S2**). We may need to consider the functional genes represented at each sampling location as well as the phylogenetic relationships. Further testing should focus on the roles of niche and traits by comparing 16S rRNA genes and functional genes within sampling locations.

Dispersal is another mechanism that could be influencing the meta-community. At the regional scale, a meta-community represents a regional pool of taxa, whereby interactions between assemblages rely on dispersal. Under the conceptual framework of Vellend (2010), ecological selection of bacterial taxa can occur in unvarying environmental conditions (with corresponding low rates of community turnover), and in variable environmental conditions (with corresponding high rates of community turnover). Depending on the concomitant rate of taxa dispersal between a pair of communities (sampling locations), the influences of a variable environment can be obscured, even when selection pressure is strong, by the homogenizing effect of a high dispersal rate that has the effect of lowering compositional differences (Vellend, 2010). Lastly, when selection pressure is weak, and the dispersal rate between communities is limiting, the dynamics of turnover within a community are subject to drift and random variations in population. Under the operational framework devised by Stegen et al. (2015) the contributions of selection, dispersal and drift were evaluated using the phylogenetic and taxonomic compositions at each sampling location relative to the meta-community. Null model expectations based on nearest taxon index (βNTI) and an extended Raup-Crick (Chase et al., 2011), referred to as RC_bray_, (Stegen et al., 2015), differentiated between these roles. These comparisons are based solely on community compositions without reference to environmental or spatial factors. The resulting matrices, **Supplementary Tables S6** and **S7**, indicate selection as a driver between sampling locations from within boreholes CR-9 and CR-18 relative to the other sampling locations. The combinations of βNTI and RC_bray_, also indicated limited dispersal and random processes as drivers governing the meta-community dynamics. The signals for selection were greater at shallow sampling locations while the influences of dispersal limitation and randomness were greater at deeper sampling locations. This is a trend also seen by Gihring et al. (2007) in the microbial communities in South African gold mines.

While the beta diversity modeling indicates a role for selection as a driver of community assembly on a regional scale, the lower taxa richness and dispersal limitation also suggest regional scale drift. Isolated communities experience a greater degree of ecological drift, and thus higher beta diversity than more connected communities (reviewed in Chase et al., 2011). A distance decay of similarity based on taxonomic (Bray–Curtis) and phylogenetic (1-UniFrac) comparisons (**Figure 5**) showed that similarity between locations leveled off at inter-location distances of up to 1.5 km. The meta-community may reflect isolation created by the rock mass and limited connectivity between fractures rather than environmental gradients (Nekola and White, 1999). The constrained RDA with forward selection of geochemical and spatial variables relative to the meta-community taxonomy identified only a spatial component in the model; accounting for 20.4% of the adjusted R^2^ variance of the pool of taxa. The environmental variables also displayed significant positive Moran’s I values (**Figure 7**) in the first spatial lag.

Overall, the spatial structure of the sampled fractures described the dynamics of the meta-community. Although individual assemblages suggest selection, the spatial structure of the sampling favored detection of limited dispersal as the main driving force governing the meta-community. The beta diversity of the study site also reflects some selection and some drift. To allow identification of variables associated with *in situ* processes of energy flow and element cycling, future groundwater studies should consider dividing the study site into smaller regions of interest with more closely spaced sampling locations or to limit sampling to hydraulically connected fractures

## Conclusion

The spatial scale of sampling to create a meta-community of suspended subsurface bacteria favored detection of neutral over selective processes. Most of the taxa identified were low abundance and represented uncultured lineages from the metabolically diverse Betaproteobacteria, Deltaproteobacteria, Bacteroidetes, Actinobacteria, Nitrospirae, and Firmicutes. Each microbial assemblage was composed of fewer taxa relative to the meta-community, but these taxa were more related than would be predicted by chance. The combination of dispersion, at one phylogenetic depth, and clustering, at another phylogenetic depth, suggest both niche (dispersion) and filtering (clustering) as drivers of local assembly.

Beta diversity also indicated selection as a driver of the subsurface meta-community; however, when attempting to relate taxa abundance to the environment, the spatial scale of groundwater sampling favored detection of neutral over selective processes. Major geochemical components were also spatially auto-correlated. Selection was detected at the level of the meta-community by βNTI that may be related to the selection that was detected within each assemblage by PD, MPD, and MNTD.

Dispersal limitation between assemblages and local selection means that the meta-community is subject to drift, and therefore, likely reflects differential historical events that have influenced the current bacterial compositions (Andam et al., 2016). Possible historical events that could shape a subsurface bacterial community include differential periods of surface water recharge, dynamics of the fractured rock mass that could release trapped pore water and alter the rock-water interactions, new fractures formed by rebound, and fractures that restricted hydraulic flow paths. A sampling design that includes more closely spaced fractures, or includes potentially hydraulically connected fractures, might reveal a contribution of environment and selection at the level of the meta-community.

## Author Contributions

All authors collected and contributed data sets for analysis as well as participated in the conceptual drafting and revision of this manuscript. KK-S oversaw the groundwater sampling and geochemical analyses. BWS and HN performed sequencing and sequence data analysis. EB conducted subsequent analyses on the sequence data and was the primary author in writing and revising the manuscript. BSS and MS contributed significantly in manuscript development and revision.

## Conflict of Interest Statement

The authors declare that the research was conducted in the absence of any commercial or financial relationships that could be construed as a potential conflict of interest.
