# Supplementary material for: Local and Regional Diversity Reveals Dispersal Limitation and Drift as Drivers for Groundwater Bacterial Communities from a Fractured Granite Formation
Source: Front Microbiol. 2016 Dec 6;7:1933. doi: 10.3389/fmicb.2016.01933 (PMC5138202; doi:10.3389/fmicb.2016.01933)
Supplement: Supplementary file 1 [file Data_Sheet_1.pdf]

Supplemental Information

The Chalk River Laboratories Study Site

The CRL site is located about 200 km northwest of Ottawa, Ontario. It is situated around the northern margin of the Ottawa-Bonnechere graben (1). Boreholes were drilled into the rock mass to assess the local geological features and then they were cased and sealed to isolate discrete fracture zones and faults to allow an assessment of the hydrogeology of the site (2, 3). The location of the site and the locations of the boreholes included in the study are shown in Figure 1. The site boundaries are formed in part by the Mattawa fault (Ottawa River) and the Maskinonge Lake fault, also shown in Figure 1. These faults may isolate groundwater into different zones across the area of study. The site is underlain by gneisses forming stacked assemblages consisting of an overlying and underlying garnet-poor assemblage (granitic and grandioritic gneiss) and a central garnet-rich assemblage (monzonitic gneiss and quartzfeldspathic gneiss) (3). Fracture infilling materials consist of magmatic intrusions (including diabase dykes of the Grenville mafic dyke swarm) and a range of low-temperature minerals dominated by chlorite and calcite as well hematite, clays and other minerals, depending upon the host rock (4). The groundwater age coincides with the opening of the North Bay outlet when overflow was diverted into the Ottawa Valley about 10,500 BP (before present)) (5).

References

1. **Kay GM.** 1942. Ottawa-Bonnechere Graben and Lake Ontario homocline. Geological Society of America Bulletin **53:**585-646.
2. **Sikorsky RI, Thivierge RH, Siddiqui J.** 2011. Geologic Characterization of the Deep Gneissic Bedrock at Chalk River Laboratories (Ontario) using Oriented Drill Core and Integrated Borehole Surveys, Canadian Nuclear Society, Waste Management, Decommissioning and Environmental Restoration for Canada's Nuclear Activities, Current Practices and Future Needs. Canadian Nuclear Society, Toronto, Canada.
3. **Thompson P, Baumgartner P, Beaton ED, Chan T, Kitson C, Kozak E, Man A, Martino JB, Sharp K, Stroes-Gascoyne S, Thivierge RH.** 2011. An Investigation of the Suitability of the Chalk River Site to Host a Geologic Waste Management Facility for AECL's Low and Intermediate Level Wastes., Waste Management, Decommissioning and Environmental Restoration for Canada's Nuclear Activities, Current Practices and Future Needs. Canadian Nuclear Society, Toronto, Canada.
4. **Dugal JJB, Kamineni DC.** 1989. Lithology, Fracture Intensity, and Fracture Filling of Drill Core From Chalk River Research Area, Ontario. *In* Thomas MD, Dixon, D. F. (ed.), Proceedings of a Workshop On Geophysical and Related Geoscientific Research At Chalk River, Ontario.
5. **Karrow PF, Anderson TW, Clarke AH, Delorme LD, Sreenivasa MR.** 1975. Stratigraphy, paleontology, and age of Lake Algonquin sediments in southwestern Ontario, Canada. Quaternary Research **5:**49-87.

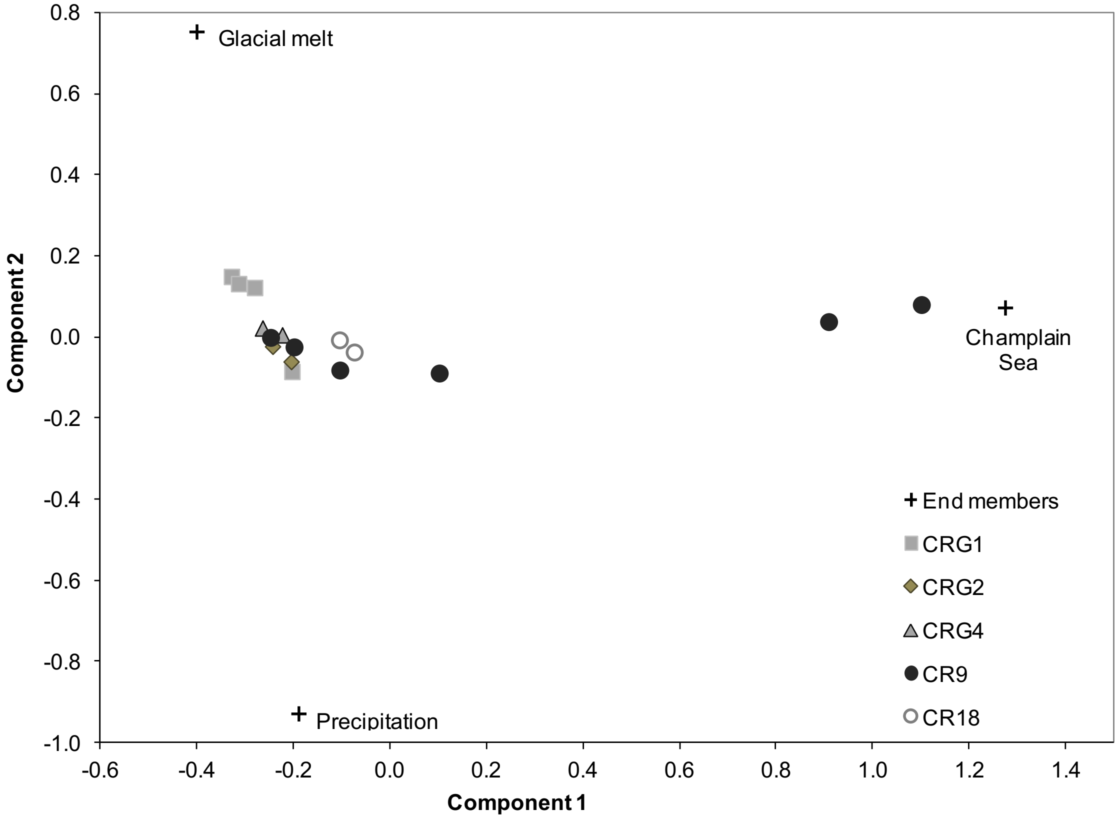

Figure S1. Principle component analysis based on major ions and stable isotopes of oxygen and hydrogen. From the Multivariate, Mixing and Mass Balance (M3) Modeling of fracture zones. CR-9 (filled circles), CR-18 (open circles), CRG-1 (squares), CRG-2 (diamonds) and CRG-4A (triangles). Compositional end members (crosses) are for glacial melt water, Champlain Sea water and modern precipitation

Table S1. Estimated concentrations[a] of bacterial 16S rRNA genes and cells ($x10^5$ per mL) in sampled groundwater.

Sample	Gene copies		Direct cell counts	
**CRG1.3**	84.02	(0.51)	1.30	(0.08)
**CRG1.6**	38.23	(5.77)	2.44	(0.48)
**CRG1.8**	65.54	(1.42)	1.77	(0.49)
**CRG-1.14**	69.36	(12.05)	4.78	(1.56)
**CRG2.2**	9.97	(0.50)	3.68	(0.34)
**CRG2.4**	10.15	(0.36)	1.14	(0.52)
**CRG4A.9**	126.11	(9.17)	11.0	(1.71)
**CR9.2**	2.33	(0.14)	0.55	(0.02)
**CR9.5**	3.04	(0.29)	0.52	(0.03)
**CR9.12**	4.27	(0.60)	0.67	(0.10)
**CR18**	73.25	(88.71)	1.94	(0.34)

[a] mean values (n = 3), standard deviation in parentheses

Table S2 – Absolute abundances of taxa by borehole and interval (link)

Table S3. Taxa Richness and Phylogenetic diversity (PD) within sampling locations.

Sampling Location		Taxa Richness	Phylogenetic Diversity, PD			
Borehole	Interval		Observed	Null ± sd	z-value	p-value
CR-18	--	221	14.7	17.5±0.4	-6.4	<0.001
CR-9	2	319	22.5	23.2±0.4	-1.7	<0.034
	5	218	16.2	17.4±0.4	-2.5	<0.001
	12	223	15.9	17.7±0.4	-4.2	<0.001
CRG-1	3	221	13.5	17.6±0.4	-9.3	<0.001
	6	253	15.8	19.5±0.4	-8.5	<0.001
	8	224	13.8	17.8±0.4	-9.3	<0.001
	14	195	11.8	16.0±0.4	-9.8	<0.001
CRG-2	2	262	15.4	20.0±0.4	-11	<0.001
	4	262	15.8	20.0±0.4	-9.6	<0.001
CRG-4A	9	180	11.3	15.0±0.4	-9.1	<0.001

Table S4. Mean pair wise distance (MPD) within sampling locations

Sampling Location		Mean Pair wise Distance (MPD				
Borehole	Interval	Observed	Null ± sd	z-value	p-value	NTI
CR-18	--	0.25	0.26 ± 0.02	-0.5	0.18	0.5
CR-9	2	0.29	0.28 ± 0.01	1.0	0.81	-1.0
	5	0.24	0.26 ± 0.02	-1.0	0.09	1.0
	12	0.26	0.27 ± 0.02	-0.5	0.23	0.5
CRG-1	3	0.22	0.23 ± 0.02	0.5	0.57	-0.5
	6	0.25	0.27 ± 0.02	-2.0	0.02	2.0
	8	0.24	0.26 ± 0.02	-1.0	0.07	1.0
	14	0.24	0.24 ± 0.02	0.0	0.47	0.0
CRG-2	2	0.25	0.24 ± 0.02	0.5	0.72	-0.5
	4	0.28	0.27 ± 0.02	0.5	0.73	-0.5
CRG-4A	9	0.24	0.26 ± 0.02	-1.0	0.19	1.0

Table S5. Mean nearest taxon distance (MNTD) within sampling locations

Sampling Location		Mean Nearest Taxon Distance, MNTD				
**Borehole**	**Interval**	**Observed**	**Null ± sd**	**z-value**	**p-value**	**NTI**
CR-18	--	0.09	0.12±0.02	-1.5	0.069	1.5
CR-9	2	0.08	0.11±0.01	-2.4	0.006	2.4
	5	0.07	0.12±0.02	-2.0	0.005	2.0
	12	0.07	0.12±0.01	-3.1	0.001	3.1
CRG-1	3	0.04	0.12±0.03	-2.9	0.001	2.9
	6	0.06	0.11±0.01	-3.6	0.001	3.5
	8	0.04	0.12±0.02	-3.8	0.001	3.8
	14	0.04	0.12±0.03	-3.1	0.001	3.1
CRG-2	2	0.06	0.12±0.01	-4.1	0.001	4.1
	4	0.05	0.11±0.02	-3.2	0.001	3.2
CRG-4A	9	0.05	0.12±0.03	-2.2	0.001	2.2

Relative Influences of Ecological Processes on the Meta-Community

Table S6. β Nearest Taxon Index distances between paired sampling locations

Borehole	CR18	CR9	CR9	CR9	CRG1	CRG1	CRG1	CRG1	CRG2	CRG2
**Interval**		**2**	**5**	**12**	**14**	**3**	**6**	**8**	**2**	**4**
**CR9.2**	3.84									
**CR9.5**	0.65	2.22								
**CR9.12**	2.41	-0.06	1.06							
**CRG1. 14**	0.03	4.90	3.28	1.36						
**CRG1. 3**	2.62	2.37	3.48	1.2	-1.39					
**CRG1. 6**	1.67	3.39	2.5	0.64	0.91	-1.46				
**CRG1. 8**	1.67	4.01	2.28	0.58	0.52	-2.39	1.51			
**CRG2. 2**	1.67	2.95	-0.46	-0.83	0.01	-1.96	0.03	-1.57		
**CRG2. 4**	2.26	3.52	-0.06	-0.97	-0.31	-0.95	2.28	-0.76	1.01	
**CRG4A. 9**	2.09	5.78	0.16	0.54	-1.11	-2.33	1.28	-0.95	-0.52	-1.54

Table S7. Raup-Crick Bray-Curtis, RC_{bray}, distances between paired sampling locations

Borehole	CR18	CR9	CR9	CR9.	CRG1	CRG1	CRG1	CRG1	CRG2	CRG2
**Interval**		**2**	**5**	**12**	**14**	**3**	**6**	**8**	**2**	**4**
**CR9.2**	1.00									
**CR9.5**	1.00	1.00								
**CR9.12**	1.00	1.00	0.04							
**CRG1.14**	1.00	1.00	1.00	1.00						
**CRG1.3**	1.00	1.00	1.00	1.00	0.60					
**CRG1.6**	1.00	1.00	1.00	1.00	0.82	0.87				
**CRG1.8**	1.00	1.00	1.00	1.00	0.46	0.59	0.56			
**CRG2.2**	1.00	1.00	1.00	1.00	0.98	1.00	1.00	0.96		
**CRG2.4**	1.00	1.00	1.00	1.00	0.99	1.00	1.00	0.99	0.48	
**CRG4A.9**	1.00	1.00	1.00	1.00	0.92	0.74	1.00	1.00	1.00	1.00

	βNTI	RC_{bray}
Selection (variable)	> +2.0	--
Selection (homogeneous)	< -2.0	--
Dispersal (limiting, drift)	null	> +0.95
Stochastic	null	null
Dispersal (homogenising)	null	< -0.95

Modelling the Meta-Community to Identify Significant Environmental and Spatial Variables

S1. Spatial variables explain taxa abundances of the meta-community

standardize the independent variables for modeling and calculate the scores of the principle components (PCA) for the environmental and spatial variables.

```
env.mem.stand=decostand(env.mem, method = "standardize")
pca.mem = principal(env.mem.stand, nfactors = 6,rotate = "none",
covar = T, scores = TRUE)
scores = pca.mem$scores # axes scores for future use
```

perform a redundancy analysis (RDA) comparing the Hellinger transformed taxa abundances with the PCA scores for the environmental and spatial variables.

```
rda.all.mem = rda(sp.hel, scores)
anova.cca(rda.all.mem)  # to check the overall explanatory power
of pca axes

     Permutation test for rda under reduced model
     Permutation: free
     Number of permutations: 999

     Model: rda(X = sp.hel, Y = scores)
              Df Variance      F Pr(>F)
     Model     6  0.43388 1.6952  0.017 *
     Residual  4  0.17063
     ---
     Signif. codes:  0 '***' 0.001 '**' 0.01 '*' 0.05 '.' 0.1 '
     ' 1

     r2.mem = RsquareAdj(rda.all.mem)
     r2.mem
          $r.squared
          [1] 0.7177373

          $adj.r.squared
          [1] 0.2943433  ##Global Adjusted R²
```

#spatial and environmental variables are significant, with a global adjusted R^2 of 29.4%.
create a reduced model to identify the significant explanatory variables.

```
mod0=rda(sp.hel~1,env.mem)  #model with intercept only
mod1=rda(sp.hel~DOC+HCO3+sulfte+iron+manganese+MEM1+MEM2+MEM3,env
.mem) #model with all explanatory variable

#Forward selection
sp.model.mem=ordistep(mod0, scope = formula(mod1),
direction="forward", perm.max = 999)
anova(sp.model.mem)
```

```
Permutation test for rda under reduced model
Permutation: free
Number of permutations: 999

Model: rda(formula = sp.hel ~ MEM1 + MEM2, data = env.mem)
         Df Variance     F Pr(>F)
Model     2  0.24644 2.753  0.001 ***
Residual  8  0.35807
---
Signif. codes:  0 '***' 0.001 '**' 0.01 '*' 0.05 '.' 0.1 '
' 1
```

```
# calculate adjusted R² for the reduced model

step.res.mem <- ordiR2step(mod0, scope = formula(sp.model.mem),
direction="forward")
step.res.mem$anova  # Summary table

                 R2.adj Df     AIC      F Pr(>F)
+ MEM1          0.20367  1 -6.2493 3.5577  0.002 **
<All variables> 0.25959
```

the spatial variables, MEM1 and MEM2 account for 25.9% of the adjusted R^2.
the spatial variable, MEM1 accounts for 20.4% of the variation. No environmental variables were identified as significant.

```
library(ade4)
library(adegraphics)
library(adespatial)
library(psych)
library(simba)
library(fossil)
library(vegan)
library(psych)
library(picante)


#load phy and com or build phy and comm form seq file
#match phy and comm in picante
#perform ses.pd and ses.mntd in picante
#perform bNTI calc and rcbray calc with Stegen's code
#normalize bNTI and RCbray -- Stegen code
#quantitative ecology distance decay calculation, with long lat
#GuniFrac has code for weighted Unifrac


#comm file tranfored with decostand specifying hellinger transformation == sp.hel
#env.mem file stadnardised by deconstand specifying stand == env.mem.stand

nbtri <- tri2nb(as.matrix(x.y[1:12,]))##Long Lat neighbour matrix
lwB <- nb2listw(nbtri, style = "B")  ##listw object
lwW <- nb2listw(nbtri, style = "W")  ##listw object, weighted
scB <- mem(lwB)
scW <- mem(lwW)
moran.bounds(lwB)


moran.randtest(env.mem[,#], listw, nrepet = 999
moranNP.randtest(x, listw, nrepet = 999, alter = c("greater", "less", "two-
sided"), ...)
scores.listw(lwW, wt = rep(1, length(lwW$neighbours)),MEM.autocor ="positive")
positiveI=scores.listw(lwW, wt = rep(1, length(lwW$neighbours)),MEM.autocor
="positive")
negativeI=scores.listw(lwW, wt = rep(1, length(lwW$neighbours)),MEM.autocor
="negative")

env$MEM1=positiveI$MEM1
env$MEM2=positiveI$MEM2
env$MEM3=positiveI$MEM3
env.mem=env[,c(1:5,11:15)]


env.mem.stand=decostand(env.mem[,-6], method = "standardize")
pca.mem = principal(env.mem.stand, nfactors = 6,rotate = "none", covar = T,
scores = TRUE)
```

```
scores = pca.mem$scores  # axes scores for future use
rda.all.mem = rda(sp.hel, scores)
anova.cca(rda.all.mem)  # to check the overall explanatory power of pca axes

r2.mem = RsquareAdj(rda.all.mem)
r2.mem$r.squared
r2mem$adj.r.squared


mod0=rda(sp.hel~1,env.mem)  #model with intercept only
mod1=rda(sp.hel~.,env.mem) #model with all explanatory variables, env and mem
sp.model=ordistep(mod0, scope = formula(mod1), direction="forward", perm.max =
999)
anova(sp.model)
step.res<- ordiR2step(mod0, scope = formula(sp.model), direction="forward")
RsquareAdj(step.res)



#repeat with env only, or with mem only

mod1.mem=rda(sp.hel~MEM1+MEM2+MEM3......etc.   ,env.mem),
sp.model.mem=ordistep(mod0, scope = formula(mod1.mem), direction="forward",
perm.max = 999)
anova(sp.model.mem),
step.res.mem <- ordiR2step(mod0, scope = formula(sp.model.mem),
direction="forward", perm.max = 999),
anova(step.res.mem), # Summary table
step.res.mem$anova,


###


mod1.env=rda(sp.hel~env1+env2......etc.   ,env.mem)
sp.model.env=ordistep(mod0, scope = formula(mod1.env), direction="forward",
perm.max = 999)
anova(sp.model.env)
step.res.env <- ordiR2step(mod0, scope = formula(sp.model.env),
direction="forward", perm.max = 999)
anova(step.res.env)  # Summary table
step.res.env$anova
```
